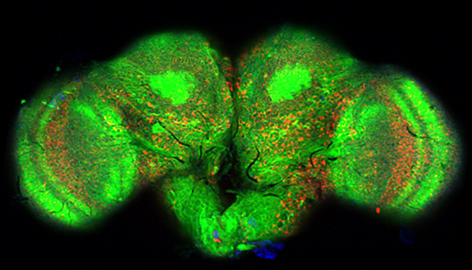# Circadian rhythmicity in a fly model of Alzheimer’s disease

**Published:** 2014-04

Sleep disruption is an early feature of Alzheimer’s disease, the commonest cause of dementia. Molecular oscillators in the central clock neurons that are entrained by light normally control human circadian rhythmicity. Here, to gain insights into sleep/wake cycle abnormalities in Alzheimer’s disease, Crowther et al. study the circadian behaviour of transgenic flies that express amyloid beta peptide (Aβ) in their neurons; extracellular amyloid plaques composed of Aβ are a characteristic of Alzheimer’s disease. Pan-neuronal expression of Aβ, the researchers report, results in progressive loss of circadian behavioural rhythmicity but does not disrupt the central molecular clock. Instead, Aβ toxicity affects communication of the rhythm to the periphery. Notably, synchronisation of the central molecular clock by exposure to light-dark cycles, although not affecting behavioural arrhythmia, prolongs the lifespan of Aβ flies, thereby supporting the use of light therapy in people with Alzheimer’s disease. Page 445

Image courtesy of Dr Stanislav Ott, University of Cambridge, Department of Genetics.

**Figure:**